# *ALK*-rearranged lung squamous cell carcinoma responding to alectinib: a case report and review of the literature

**DOI:** 10.1186/s12885-017-3468-1

**Published:** 2017-07-06

**Authors:** Nobuaki Mamesaya, Kazuhisa Nakashima, Tateaki Naito, Takashi Nakajima, Masahiro Endo, Toshiaki Takahashi

**Affiliations:** 1 Divisions of Thoracic Oncology, Shizuoka Cancer Center, 1007 Shimonagakubo, Nagaizumi-cho, Sunto-gun, Shizuoka, 411-8777 Japan; 2 Divisions of Pathology, Shizuoka Cancer Center, Shizuoka, Japan; 3 Divisions of Diagnostic Radiology, Shizuoka Cancer Center, Shizuoka, Japan

**Keywords:** Alectinib, Anaplastic lymphoma kinase, Lung squamous cell carcinoma

## Abstract

**Background:**

Although anaplastic lymphoma kinase (*ALK*) fusion genes are generally identified in lung adenocarcinoma patients, they are relatively rare in patients with squamous cell carcinoma (SqCC). Metastatic *ALK*-rearranged lung adenocarcinoma patients treated with ALK inhibitors demonstrate higher response rates, improved progression-free survival, and reduced toxicity relative to those treated with conventional chemotherapy regimens. However, the efficacy of treatment with ALK inhibitors in patients with *ALK*-rearranged lung SqCC remains unknown.

**Case presentation:**

We discuss a 52-year-old Japanese-Brazilian woman without a history of smoking who was referred to our hospital for evaluation of severe left back pain and a left hilar mass observed on a chest radiograph. The patient was eventually diagnosed on the basis of computed tomography, pathological, and immunohistochemical findings as having Stage IV lung SqCC. First-line treatment with palliative radiotherapy and systemic chemotherapy with cisplatin plus vinorelbine was administered, but was not effective. *ALK* testing was subsequently performed, revealing positive *ALK* expression and gene rearrangement. Alectinib therapy was then initiated, which resulted in a gradual, but substantial reduction in tumor size.

**Conclusions:**

To the best of our knowledge, this is the first case report to discuss the successful management of *ALK*-rearranged lung SqCC with alectinib. We propose that molecular testing for driver mutations should be considered in young patients with a light or no smoking history, even if the histological findings correspond with SqCC, and alectinib therapy represents a reasonable option in cases of *ALK*-rearranged lung SqCC.

## Background

Numerous oncogenic driver mutations have been identified in patients with non-small cell lung cancer. Research has reported that two genes in particular (human epidermal growth factor receptor [*EGFR*] and anaplastic lymphoma kinase [*ALK*]) are associated with improvements in therapeutic efficiency in non-small cell lung cancer patients receiving targeted therapies. *ALK* fusion genes are typically identified in approximately 5.0% of patients with lung adenocarcinoma, although they are rare in patients with squamous cell carcinoma (SqCC) [1, 2]. Treatment of metastatic *ALK*-rearranged non-small cell lung cancer with ALK inhibitors leads to higher response rates and improved progression-free survival relative to conventional chemotherapy regimens [3, 4]. However, the efficacy of such treatment for *ALK*-rearranged lung SqCC remains unknown, as *ALK*-rearranged lung SqCC is very rare and *ALK* testing is not routinely performed in this patient population. Herein, we describe a rare case of *ALK*-rearranged lung SqCC responding to alectinib.

## Case presentation

**Fig. 1:**
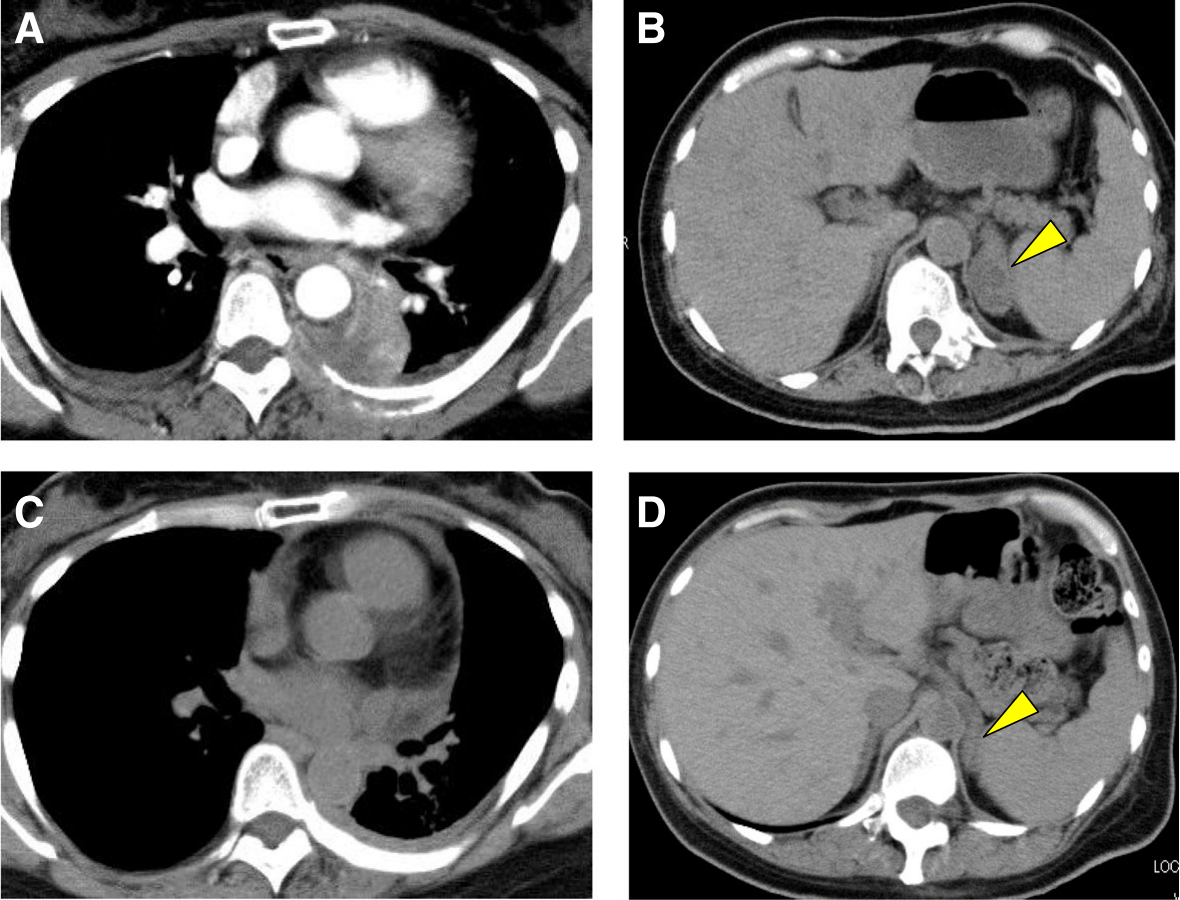
Computed tomography findings before and after treatment with alectinib. A computed tomography scan before treatment revealed (**a**) a solitary tumor in the lower lobe of the left lung and (**b**) a left adrenal metastasis (*arrow*). A computed tomography scan 3 months after commencing treatment revealed (**c**) a dramatic reduction in tumor size and (**d**) almost no presence of metastases in the left adrenal gland (*arrow*)

**Fig. 2:**
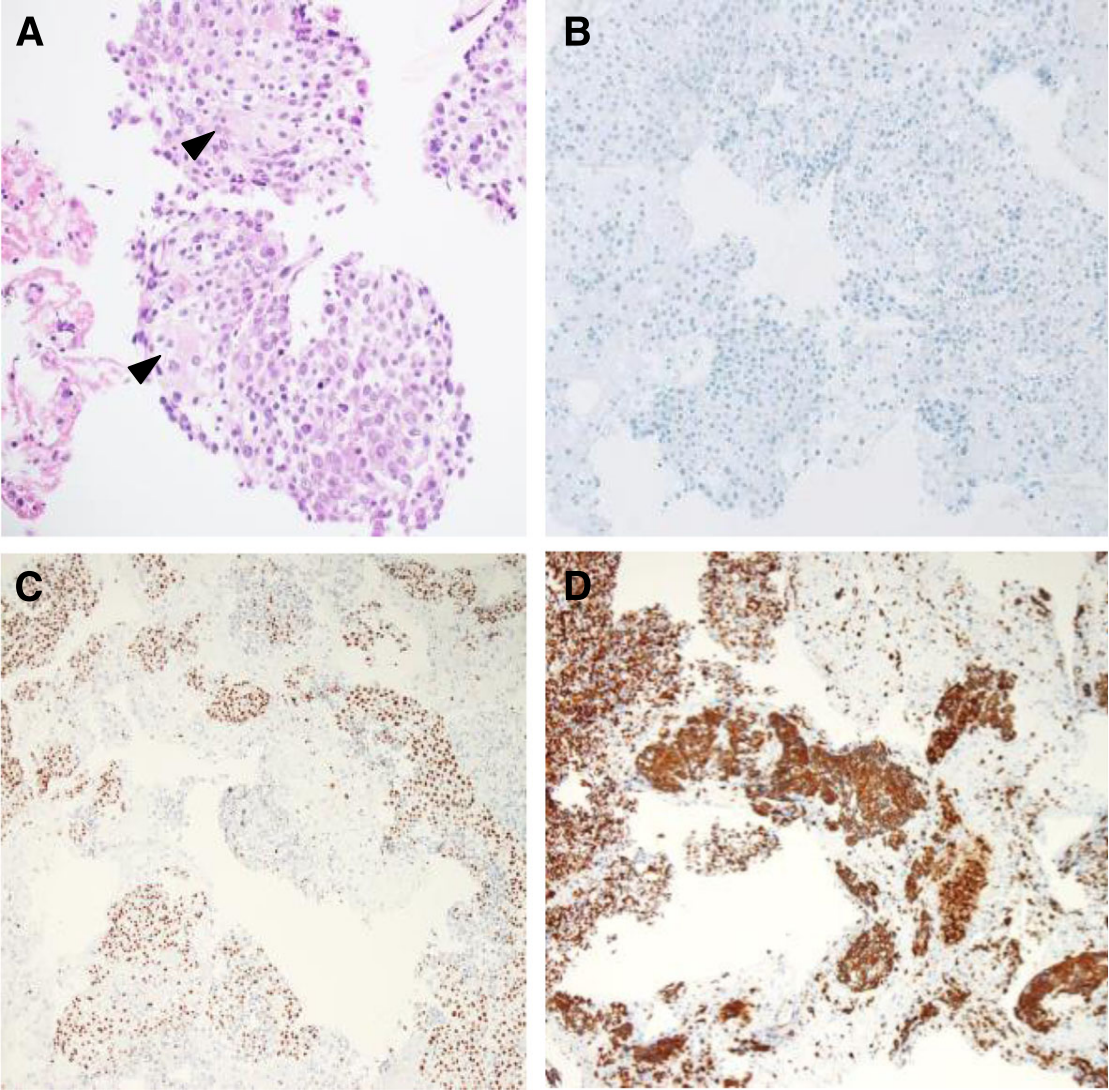
Transbronchial lung biopsy specimen from a mediastinal lymph node. **a** Hematoxylin and eosin staining revealed undifferentiated cancer cells with a mild tendency of cornification (*arrows*). Immunohistochemical staining revealed that the tumor cells were negative for (**b**) thyroid transcription factor-1, but positive for (**c**) p40 and (**d**) cytokeratin 5/6

**Fig. 3:**
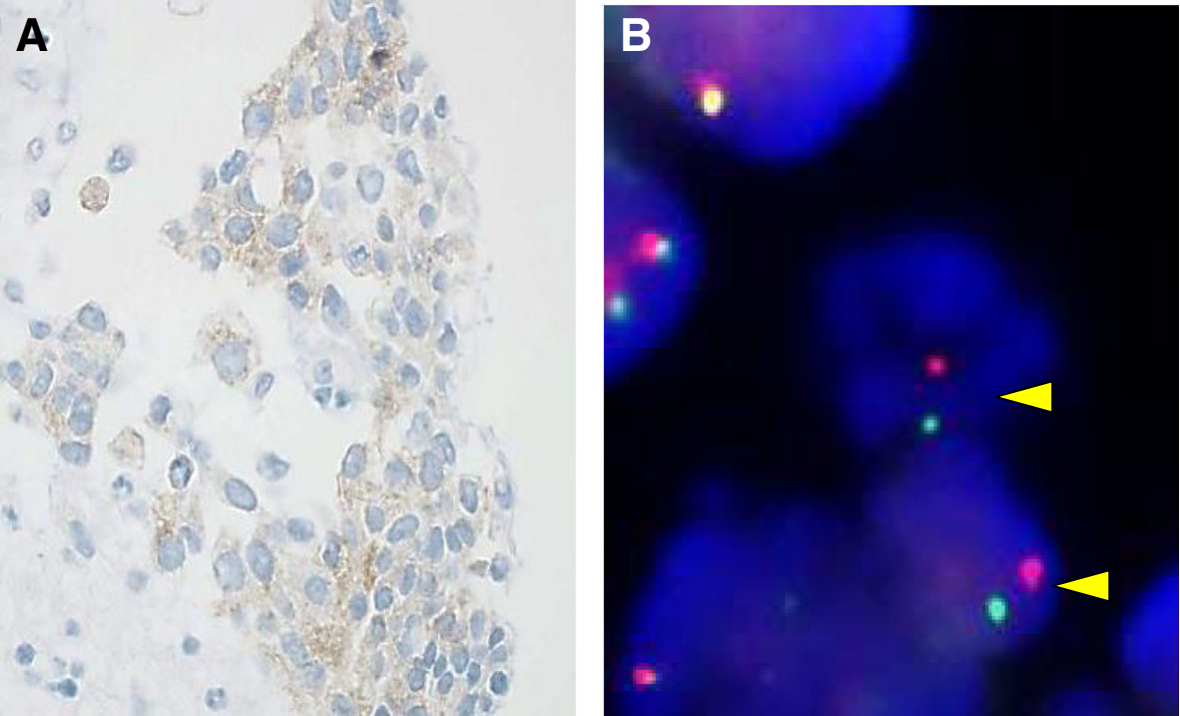
Anaplastic lymphoma kinase testing. **a** Immunohistochemical analysis revealed anaplastic lymphoma kinase positive protein expression. **b** Fluorescence in situ hybridization revealed a deletion of the 5′ signal with retained 3′ signal (*arrows*), consistent with an anaplastic lymphoma kinase rearrangement

A 52-year-old Japanese-Brazilian woman without a history of smoking was referred to our hospital for evaluation of severe left back pain and a left hilar mass observed on a chest radiograph. Computed tomography of the chest revealed a solitary tumor in the left lower lobe with direct invasion to the seventh thoracic vertebra and rib. The patient also had mediastinal lymphadenopathy, left adrenal metastasis, and multiple bone metastases (Fig. 1a-b). Pathological examination of the transbronchial needle aspiration biopsy specimen revealed undifferentiated cancer with a mild tendency of cornification (hematoxylin and eosin staining, Fig. 2a). Upon immunohistochemical (IHC) analysis, the tumor cells exhibited strong positive staining for p40 and cytokeratin 5/6, but were negative for thyroid transcription factor-1 (Fig. 2b-d). Based on these findings, the patient was diagnosed with Stage IV lung SqCC and was treated with palliative radiotherapy and first-line systemic chemotherapy with cisplatin plus vinorelbine. After 2 cycles of chemotherapy, there was no evidence of a response. Second-line chemotherapy was thus indicated. Despite a diagnosis of SqCC, the patient underwent *ALK* testing, as she was a non-smoker diagnosed with lung cancer harboring the wild-type *EGFR* gene. IHC analysis indicated that the tumor cells were positive (2+ staining) for the ALK antibody (Histofine ALK iAEP Detection Kit; Nichirei Bioscience Inc., Tokyo, Japan). ALK break-apart fluorescence in situ hybridization (FISH) (Vysis ALK Break Apart FISH Probe Kit; Abbott Molecular, Inc., Des Plaines, IL, USA) confirmed the presence of an *ALK* gene rearrangement with a rearrangement-positive cell rate of 46.0% (Fig. 3). The patient was subsequently treated with alectinib, a selective ALK inhibitor. After 2 weeks of treatment, the symptoms gradually improved. After 3 months, a follow-up computed tomography scan revealed a remarkable response in the primary lesion and significant shrinkage of the left adrenal gland metastasis (Fig. 1c-d). At the latest follow-up, 11 months after commencing alectinib treatment, there was no evidence of progression or any remarkable toxicity.

## Discussion and conclusions

To the best of our knowledge, this is the first case report to discuss the successful management of *ALK*-rearranged lung SqCC with alectinib.

Our diagnosis of SqCC was confirmed by p40 immunostaining, which is useful and highly specific for the diagnosis of SqCC [5, 6]. Additionally, in our case, the results of the *ALK* detection test were concordant between IHC staining and FISH. Yamamoto et al. [7] reported a similar case, which was diagnosed with *ALK*-rearranged lung SqCC. The diagnosis was confirmed by IHC staining, which was positive for p40, but negative for thyroid transcription factor-1. The pathological specimen of their case [7] was also obtained from the primary lesion by bronchoscopic biopsy and no adenocarcinoma component was detected in the biopsy specimen. The case reported by Yamamoto et al. [7] was positive on FISH with a rearrangement-positive cell rate of just 20.0%, but negative on IHC staining. Ilie et al. [8] reported that cases with discordant *ALK* detection test results (i.e., FISH positive, but IHC staining negative) had lower rearrangement-positive cell rates of 15.0–20.0% and exhibited a tendency towards a lower response to crizotinib. However, since the case described by Yamamoto et al. [7] was treated with radiotherapy without chemotherapy, it remains unclear whether the patient exhibited a marked response to ALK targeted therapies. As shown in Table 1, only a few cases of *ALK*-rearranged lung SqCC responding to crizotinib have been reported to date [9–12]. Alectinib is a new drug that is expected to be safer and more effective than crizotinib as a first-line chemotherapy treatment for patients with *ALK*-rearranged lung adenocarcinoma [13]. Recently, Tamiya et al. [14] reported a case of *ALK*-rearranged lung SqCC that was treated with alectinib, although no response was observed. In our case, the patient with *ALK*-rearranged lung SqCC exhibited an antitumor response to alectinib. Further case reports are needed to confirm the efficacy of ALK targeted therapies for the treatment of *ALK*-rearranged lung SqCC patients.

**Table 1:** Literature review of all clinical cases to date

Authors	Age (y)	Sex	Method of diagnosis and/or type of tissue sampled	ALK detection	Smoking history (pack-years)	Prior treatment	ALK inhibitor	Efficacy
Wang et al. [9]	55	F	Biopsy of the cervical lymph node	IHC, FISH	Non-smoker	PDC	Crizotinib	PR
Mikes et al. [10]	36	M	Bronchial biopsy of the primary lesion	IHC, FISH, RT-PCR	Non-smoker	None	Crizotinib	PR
Zhang et al. [11]	55	F	Bronchial biopsy of the primary lesion	IHC	Non-smoker	PDC	Crizotinib	PR
Vergne et al. [12]	58	F	Bronchial biopsy	IHC, FISH	Non-smoker	PDC	Crizotinib	PR
Tamiya et al. [13]	78	M	Primary lesion	IHC, FISH	49	None	Alectinib	PD
This case	52	F	Bronchial biopsy of the mediastinal lymph node	IHC, FISH	Non-smoker	PDC	Alectinib	PR

*ALK* anaplastic lymphoma kinase, *F* female, *FISH* fluorescence in situ hybridization, *IHC* immunohistochemistry, *M* male, *PCR* polymerase chain reaction, *PD* progressive disease, *PDC* platinum-doublet chemotherapy, *PR* partial response, *RT* reverse transcription, *y* year

There are some limitations to our case report. Our histological specimen was small and was obtained from a mediastinal lymph node. For this reason, there was the potential for an adenocarcinoma component to be contained in other regions or for there to be discrepancies between the primary lesion and metastatic lesions due to the heterogeneity and distribution of the tumor. In contrast, Hou et al. [15] reported a high concordance rate of *ALK* rearrangement between primary tumors and paired metastatic lymph nodes, which supports the findings of our case report.

In conclusion, molecular testing for driver mutations should be considered in young patients with a light or no smoking history, even if the histological findings correspond with SqCC. Alectinib represents a reasonable option in cases of *ALK*-rearranged lung SqCC.

## Abbreviations

*ALK*: Anaplastic lymphoma kinase
*EGFR*: Epidermal growth factor receptor
FISH: Fluorescence in situ hybridization
IHC: Immunohistochemical
SqCC: Squamous cell carcinoma
